# Dramatic Coronary Artery Aneurysm Regression After Coronary Artery Bypass Grafting and Proprotein Convertase Subtilisin/Kexin Type 9 Inhibitor Therapy

**DOI:** 10.1093/icvts/ivaf272

**Published:** 2025-11-17

**Authors:** Yuichiro Yokoyama, Hiroshi Higashino, Hiroyuki Fujieda, Mitsunori Abe

**Affiliations:** Yotsuba Circulation Clinic, Ehime 790-0062, Japan; Yotsuba Circulation Clinic, Ehime 790-0062, Japan; Yotsuba Circulation Clinic, Ehime 790-0062, Japan; Yotsuba Circulation Clinic, Ehime 790-0062, Japan

**Keywords:** PCSK9, coronary artery aneurysm, coronary artery bypass grafting

## Abstract

Proprotein convertase subtilisin/kexin type 9 (PCSK9) inhibitors offer clinical benefits by reducing low-density lipoprotein cholesterol levels and have emerged as valuable therapeutic agents for the management of cardiovascular diseases. Although their effectiveness in treating coronary artery plaques and abdominal aortic aneurysms has been reported, data on their effects on coronary artery aneurysms are limited. Herein, we report the case of a 43-year-old man with familial hyperlipidemia who presented with angina pectoris caused by a giant right coronary artery aneurysm accompanied by extensive plaques and severe stenosis. The patient underwent coronary artery bypass grafting at 6 anastomotic sites, and a PCSK9 inhibitor was initiated postoperatively. Five years after surgery, imaging demonstrated a gradual reduction in the size of the coronary artery aneurysm, and 8 years later, the size was further reduced. This case report illustrates the rare clinical course of a coronary artery aneurysms.

## INTRODUCTION

The major causes of coronary artery aneurysms are Kawasaki disease in children and atherosclerotic disease in adults. While spontaneous regression due to the resolution of inflammation, or treatment with immunoglobulin for Kawasaki disease, has been reported, few reports exist on the regression of atherosclerotic coronary artery aneurysms. Proprotein convertase subtilisin/kexin type 9 (PCSK9) inhibitors have been shown to prevent plaque formation; however, no study has reported that PCSK9 reduces aneurysm size. Here, we report a case in which a PCSK9 inhibitor was initiated after coronary artery bypass grafting (CABG) for three-vessel coronary artery disease complicated by a right coronary artery aneurysm. A marked reduction in aneurysm size was observed during follow-up.

## CASE REPORT

**Figure 1.**
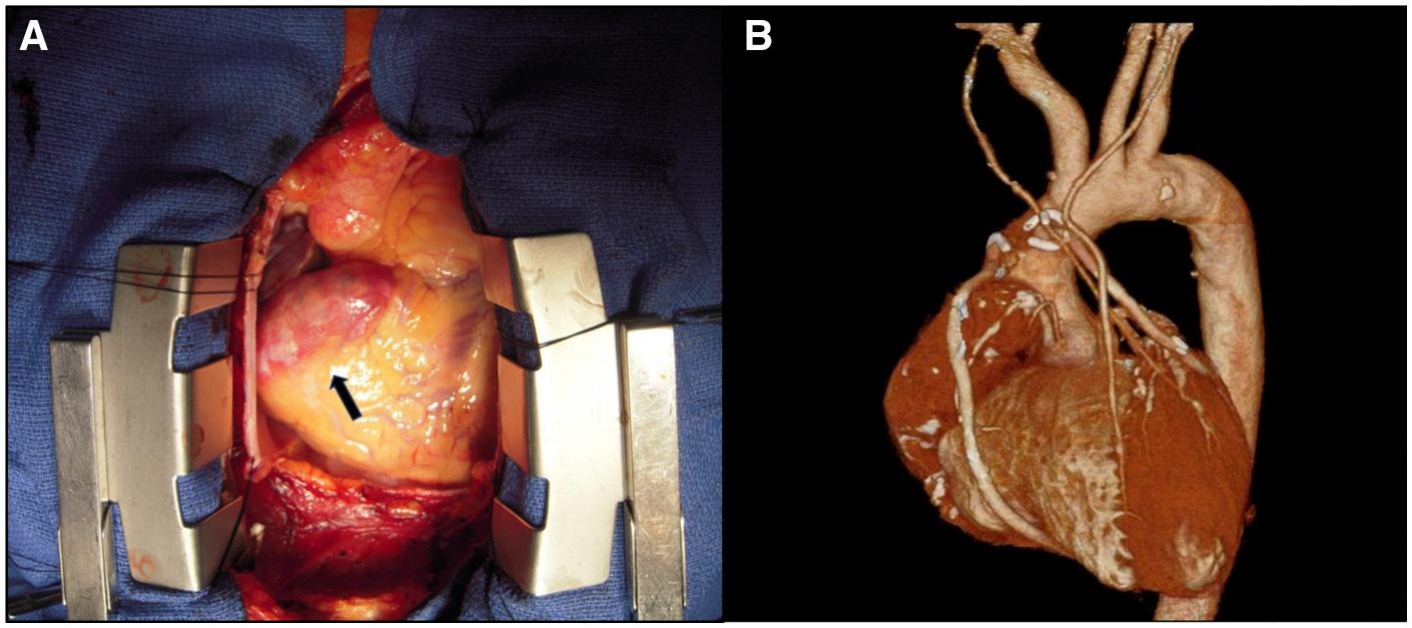
(A) Intraoperative Image Showing a Giant Right Coronary Artery Aneurysm (Arrow). (B) Computed Tomography Finding of the Coronary Artery Bypass Grafting

A 43-year-old man was referred to our clinic after a medical checkup revealed a coronary artery aneurysm. Laboratory examinations revealed markedly elevated low-density lipoprotein cholesterol (LDL) levels (382 mg/dl) and xanthomas in the elbow and knee. The patient was diagnosed with untreated familial hypercholesterolaemia (FH), a giant right coronary artery aneurysm measuring up to 30 mm in diameter (Figure 1A), and severe 3-vessel coronary artery disease. He had no medical history or physical characteristics suggestive of a connective tissue disorder, and no inflammatory response was observed in the blood test indicative of vasculitis. Percutaneous catheter intervention was deemed unsuitable, and CABG was scheduled for the patient. He underwent an uneventful CABG at 6 anastomotic sites using the bilateral internal mammary arteries and great saphenous veins (Figure 1B). High-intensity statin therapy was initiated immediately postoperatively; however, due to persistently high LDL levels, PCSK9 inhibitor therapy was added 4 months postoperatively. Low-density lipoprotein cholesterol levels decreased thereafter, but gradually began to rise again. Ezetimibe was added to the statin and PCSK9 regimens starting 3 years postoperatively, after which the LDL levels stabilized between 30 and 60 mg/dl. No interventions beyond lipid-lowering therapy, such as anti-inflammatory drugs or major lifestyle changes, were implemented during the treatment period.

**Figure 2.**
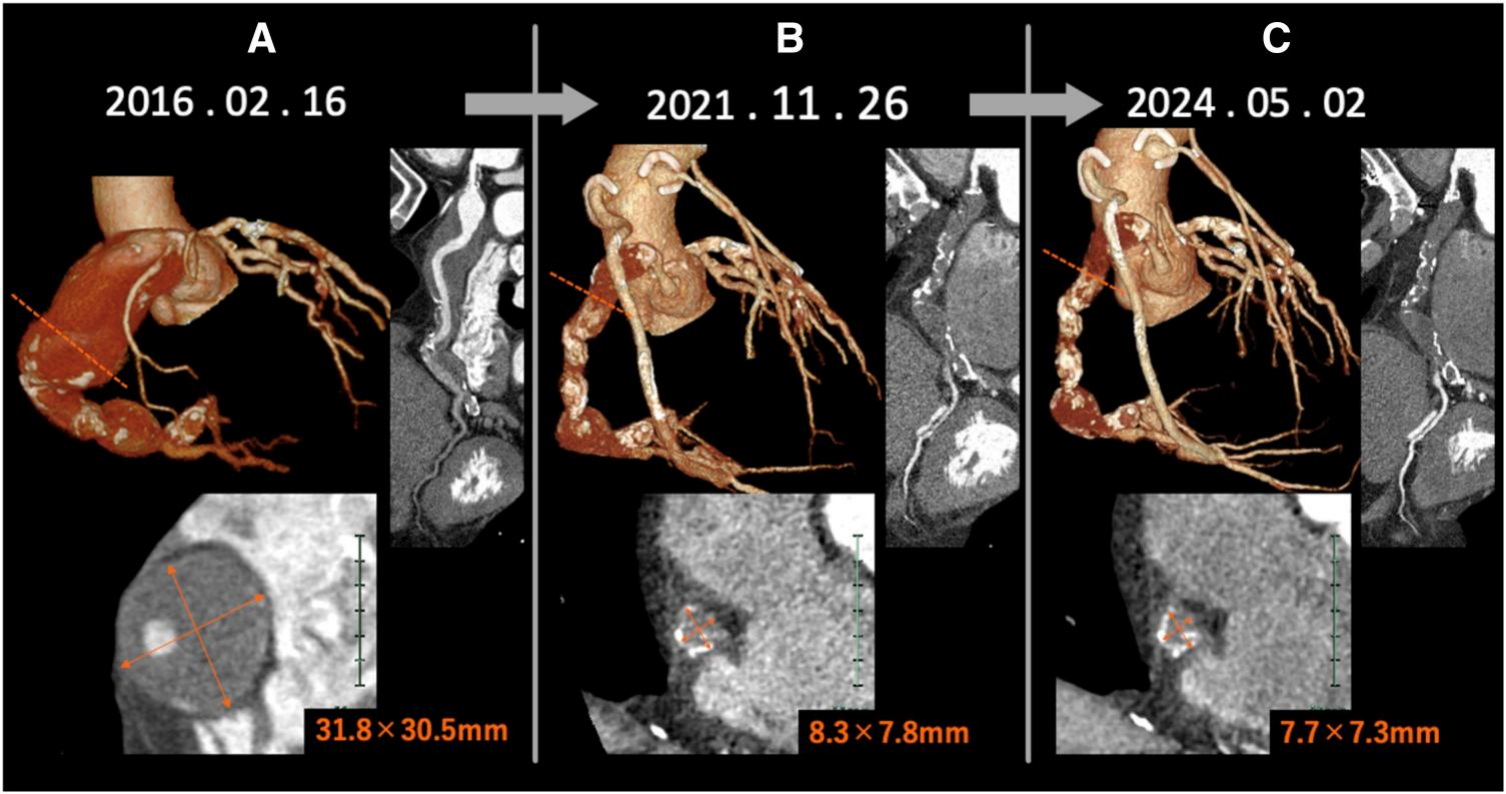
Computed Tomography Findings Illustrating Progressive Aneurysm Size Reduction over Time; (A) Preoperative, (B) Five Years Postoperative, and (C) Eight Years Postoperative.

At the fifth-year follow-up, coronary computed tomography revealed occlusion of the right coronary aneurysm and a reduction in the maximum diameter from 30 to 8 mm (Figure 2A and B). By the eighth year, further size reduction of the aneurysm was observed, along with marked plaque regression (Figure 2C). The patient remained asymptomatic and healthy without any chest complaints.

## DISCUSSION

The aetiology of giant coronary artery aneurysm is not completely understood. Atherosclerosis is the most common cause in adults, accounting for up to 50% of all cases. In paediatric populations, conditions such as Kawasaki disease and Takayasu arteritis are the primary etiologies. FH is a genetic disorder characterized by severe hypercholesterolaemia and has been implicated in the formation of coronary artery aneurysms. In addition to statins and ezetimibe, the administration of PCSK9 inhibitors is recommended. PCSK9 plays a key role in the pathogenesis of LDL receptor-mediated atherosclerosis. In addition to lipid metabolism, PCSK9 has been shown to contribute to atherosclerosis through various mechanisms, such as monocyte infiltration into plaques, macrophage-driven inflammation, and accumulation of oxidized LDL. It also influences vascular smooth muscle cell differentiation, proliferation, and migration, further contributing to its proatherogenic role in the arterial wall [1]. The 2024 EACTS guidelines on perioperative medication in adult cardiac surgery recommend introducing PCSK9 if LDL levels do not reach the target levels (<55 mg/dl or 1.4 mmol/l) despite statin and ezetimibe therapy (Class 1) [2]. This case involved FH that required intensive LDL control and reached the guideline-recommended target values. Although the mechanism by which this coronary aneurysm shrinks is multifactorial and difficult to pinpoint definitively, it is thought to reflect thrombus formation within the aneurysm due to blood flow diversion caused by bypass surgery, combined with the reduction in vascular inflammation, improvement in endothelial function, and plaque stabilization achieved by introducing PCSK9 inhibitors. However, the latest report (NEWTON-CABG CardioLink-5) trial indicates that LDL-lowering therapy with evolocumab does not contribute to SVG disease following CABG surgery, leaving the precise mechanism in this case unclear [3]. A causal relationship between PCSK9 and abdominal aortic aneurysms (AAA) has recently been reported [4, 5]. PCSK9 expression has been found to be upregulated in fibroblasts located in the neck of AAA, contributing to the weakening of the aneurysm wall [4]. In an animal study, strong PCSK9 expression, similar to that in humans, was confirmed in the aneurysm walls of an AAA model. The suppression of AAA formation or progression through a cholesterol-independent response to PCSK9 inhibitors has also been reported [5]. Similar to the findings in AAA, PCSK9 expression may also be elevated in coronary artery aneurysms, suggesting that PCSK9 inhibitors may play a role in the prevention or regression of atherosclerotic coronary artery aneurysms. To elucidate this mechanism, clinical studies should introduce PCSK9 monotherapy in patients with atherosclerotic coronary artery aneurysms without ischaemia to clarify its efficacy.

## Data Availability

The data supporting the findings of this study are available from the corresponding author, Yuichiro Yokoyama, upon reasonable request.
